# SRC-1 Knockout Exerts No Effect on Amyloid β Deposition in APP/PS1 Mice

**DOI:** 10.3389/fnagi.2020.00145

**Published:** 2020-06-17

**Authors:** Qiong Wu, Bin Wang, Qi-Fa Li, Xuan Zhang, Michael Ntim, Xue-Fei Wu, Na Li, Dan-Dan Zhu, Rong Jiang, Jin-Yi Yang, Yu-Hui Yuan, Shao Li

**Affiliations:** ^1^Liaoning Provincial Key Laboratory of Cerebral Diseases, Department of Physiology, College of Basic Medical Sciences, Dalian Medical University, Dalian, China; ^2^National-Local Joint Engineering Research Center for Drug-Research and Development (R and D) of Neurodegenerative Diseases, Dalian Medical University, Dalian, China; ^3^Department of Urology, Affiliated Dalian Friendship Hospital of Dalian Medical University, Dalian, China; ^4^Institute of Cancer Stem Cell, Dalian Medical University, Dalian, China

**Keywords:** steroid receptor coactivator 1, Alzheimer’s disease, amyloid-β, synapse, glia

## Abstract

Steroid receptor coactivator 1 (SRC-1) is the key coactivator because of its transcriptional activity. Previous studies have shown that SRC-1 is abundant in the hippocampus and has been implicated in cognition. SRC-1 is also related to some major risk factors for Alzheimer’s disease (AD), such as a decline in estrogen and aging, however, whether SRC-1 is involved in the pathogenesis of AD remains unclear. In this study, we established SRC-1 knockout in AD mice by cross breeding SRC-1^−/−^ mutant mice with APP/PS1 transgenic mice, and investigated the expression of some synaptic proteins, the amyloid β (Aβ) deposition, and activation of astrocytes and microglia in the hippocampus of APP/PS1×SRC-1^−/−^ mice. The results showed that SRC-1 knockout neither affects the Aβ plaque and activation of glia, nor changes the expression of synaptic proteins in AD model mice. The above results suggest that the complete deletion of SRC-1 in the embryo exerts no effect on the pathogenesis of APP/PS1 mice. Nevertheless, this study could not eliminate the possible role of SRC-1 in the development of AD due to the lack of observation of other events in AD such as tau hyperphosphorylation and the limitation of the animal model employed.

## Introduction

Alzheimer’s disease (AD), the most prevalent cause of dementia, is characterized by progressive cognitive impairment (Fernandez et al., 2019; Lu et al., 2019). The mechanisms of sporadic AD (representing more than 95% of AD cases) are uncertain, but some major risk factors have been identified. The incidence of AD rises exponentially with age (Villemagne et al., 2013; Wattmo and Wallin, 2017). The sex hormone offers protective effects on the brain and the deprivation during menopause or andropause triggers neurodysfunction, neurodegeneration, and cognitive disease (Meng et al., 2010; Ross et al., 2017) and disproportionately increases the risk of AD (Zhao et al., 2015; Tang et al., 2018).

Steroids, such as estrogen and androgen, have profound roles in the hippocampal function and may causally be involved in cognitive deficits in AD (Hasanpour et al., 2018). Steroid receptor coactivator 1 (SRC-1) is the key coactivator to the nuclear receptors (NRs) of the steroids and is important to their effect on transcriptional activities (Bayele, 2019; Heck et al., 2020). It has been reported that SRC-1 is abundantly expressed in specific brain regions such as the hippocampus and cortex, which are recognized as the crucial brain structures in cognition (Charlier et al., 2005, 2013; Qiu et al., 2016; Zalachoras et al., 2016). Previous studies have shown that SRC-1 is in involved in the estrogen receptor (ER) or androgen receptor (AR) induced memory formulation and synaptic plasticity in the hippocampus (Bian et al., 2012; Liu et al., 2015; Qiu et al., 2016; Zhao et al., 2017). The aged-related decrease of SRC-1 expression is also observed, especially in the hippocampus region (Zhang et al., 2011; Zhao et al., 2017). Taken together, these studies indicate that SRC-1 might be implicated in the interaction of these risk factors and the development of AD, which leads to the question of whether SRC-1 is involved in the pathogenesis of AD or not.

In this study, we first evaluated the SRC-1 level in APP/PS1 transgenic mice, and then investigated the effect of SRC-1 in the pathogenesis of AD mice by cross breeding the SRC-1 knockout (SRC-1^−/−^) mice with the APP/PS1 mice. Our results showed that SRC-1 expression in the APP/PS1 mice was not different from the wild type (WT) mice and SRC-1 deletion had no effect on Aβ deposition, activation of microglia and astrocytes, and synaptic protein expression in APP/PS1 mice. These negative results suggest that SRC-1 does not interfere with AD progression, at least in the current experimental condition.

## Materials and Methods

### Animals

SRC-1^+/−^ mice (with a C57BL/6J background) were kindly provided by Dr. Jianming Xu from Baylor College of Medicine, TX, USA. APPswe/PS1Δe9 (APP/PS1) mice (with a C57BL/6J background) were purchased from Jackson Laboratory. C57BL/6J were provided by the Animal Center of Dalian Medical University (DMU).

Male SRC-1^+/−^ were bred with SRC-1^+/−^ females. Offspring males homozygous for the SRC-1^−/−^ (SRC-1 knockout, KO) and the WT littermates (SRC-1^+/+^) were used in the experiments at 3–4 months of age.

APP/PS1 mice were bred with WT C57BL/6J. Male offspring heterozygous for the APP/PS1 transgenic construct and the WT littermates (not expressing any transgene) were used as the control. APP/PS1 mice develop plaque deposition by 6 months of age, while the plaque would be clearly visible when the mice are more than 7-months old. Male APP/PS1 and the WT littermates used in the experiments were at 8–9 months old.

All the animals were raised and bred in the Animal Center of DMU and allowed access to food and water *ad libitum*. Animals were maintained on a 12:12 light–dark cycle (lights on at 08:00, lights off at 20:00), within a temperature-controlled room (T: 24°C ± 1°C). All tests were performed during the light cycle. All experiments were carried out under the guidelines of the National Institutes of Health Guide for the Care and Use of Laboratory Animals.

### Generation of APP/PS1×SRC-1^−/−^ Mice

SRC-1 knockout in APP/PS1 (APP/PS1×SRC-1^−/−^) mice were obtained by breeding SRC-1^−/−^ mice with APP/PS1 mice, using a three-generation breeding scheme (Supplementary Figure S1). APP/PS1 mice were crossed with SRC-1^−/−^ mice to introduce the SRC-1 knockout allele into the F1 generation (APP/PS1×SRC-1^+^). APP/PS1×SRC-1^+/−^ mice were then crossed with SRC-1^+/−^ mice to obtain the F2 generation which could comprise APP/PS1×SRC-1^+/+^ (APP/PS1), APP/PS1×SRC-1^+/−^, APP/PS1×SRC-1^−/−^, SRC-1^+/+^ (WT), SRC-1^+/−^ and SRC-1^−/−^. The proportions of each genotype of the mice are shown in Supplementary Figure S1.

APP/PS1×SRC-1^−/−^ mice were then age-matched with APP/PS1×SRC-1^+/+^ mice, which were used as the control. The SRC-1^+/+^ littermates express neither the APP/PS1 gene nor SRC-1 mutants and could be considered as wildtype (WT). Female mice were only used for the breeding. All the male mice used in the experiment were 8–9 months old.

### Western Blot

The proteins were extracted using an extraction kit (Keygen Biotech, China), and the protein content was measured by a BCA protein assay (Keygen Biotech, China). For Western Blotting, the proteins (10–30 μg) for each sample were loaded into a 10% SDS PAGE gel for electrophoresis, then the protein components were transferred to polyvinylidene difluoride (PVDF) membranes. Later, the PVDF membranes were blocked with 5% bull serum albumin (BSA) in TBST (TBS + 0.1% Tween-20) for 1 h, and immunoblotted overnight at 4°C with the primary antibodies: SRC-1 (180 KD, 1:1,000, Cell signaling technology, Millipore, 2191S); PSD95 (1:1,000, Abcam, ab2723); Synapsin (70 KD, 1:1,000, Abcam, ab64581); GluR1 (100 KD, 1:1,000, Abcam, ab31232); APP (87 KD, 1:1,000, Abcam, ab15272); beta-site APP-cleaving enzyme 1 (BACE-1; 70 KD, 1:1,000, Abcam, ab2077); β-actin (1:2,000, Abcam, ab6276). The following day, the membranes were washed in TBST three times, then incubated with a horseradish peroxidase-labeled secondary antibody (anti-mouse or anti-rabbit, 1:5,000; ZSJQ-BIO Company, Beijing, China) for 1 h at room temperature. The infrared band signals were detected using BIO-RAD gel analysis software (Hercules, CA, USA). The densitometric analysis of immunoreactivity was conducted using the NIH ImageJ software (Li et al., 2019).

### Immunohistochemistry (IHC) and Immunofluorescence (IF)

IHC and IF were carried out according to our previous work (Wang et al., 2018). The primary antibodies: SRC-1 (1:100–200); 6E10 (for Aβ; 1:100, Covance, S39320260); GFAP (1:200, DAKO, 20334); Iba-1 (1:200, WAKO, 019-19741) were used. The mice were anesthetized with pentobarbital (50 mg/kg, 0.05 ml/10 g body weight, i.p.) and perfused with 0.1% phosphate buffer (PB), followed by 4% paraformaldehyde (PFA) dissolved in 0.1% PB. Afterward, the brains were removed and left in 4% PFA at 4°C for 24 h, and then transferred to 30% sucrose dissolved in 0.1% PB. Following saturation of the brains in sucrose, serial 16 μm coronal sections were made with a cryostat (Leica CM 3050 S, Leica Microsystems AG, Wetzlar, Germany) after OCT embedding. The slices that contained cortex and ventral hippocampus were used for the staining. For the IHC staining procedure, the slices were thoroughly rinsed in 0.3% PBS-T for 15 min, then quenched by 3% H_2_O_2_ in 0.01 M PBS for 15 min. Subsequently, they were rinsed again and pre-incubated in 2% BSA and 0.3% Triton X-100 in 0.01 M PBS at room temperature for 1 h, and then incubated at 4°C with the primary antibody in 0.01 M PBS containing 2% BSA and 0.3% Triton X-100 overnight. After incubation with a biotinylated goat anti-rabbit or anti-mouse IgG secondary antibody (1:200; Vector Laboratories, Burlingame, CA, USA) for 2 h, the bound antibodies were visualized using an avidin–biotin–peroxidase complex system (Vectastain ABC Elite Kit, Vector Laboratories, Burlingame, CA, USA) and then stained with diaminobenzidine (DAB; Vectro Laboratories) as a chromogen. The slides were visualized with a microscope and digitally photographed (Pannoramic Digital Slide Scanners, 3DHISTECH, Budapest, Hungary).

For the IF staining procedure, the sections were washed with 0.3% PBST (three times, 10 min each time). After that, the sections were blocked with 5% BSA in PBS containing 0.3% Triton X-100 at room temperature for 1 h, and then incubated with primary antibody overnight at 4°C. On the second day, the sections were incubated with secondary antibody for 2 h at room temperature after being washed with 0.3% PBST three times. Images were captured under a microscope (Pannoramic Digital Slide Scanners, 3DHISTECH, Budapest, Hungary). The analysis of mean integrated optical density (MIOD) was performed using ImageJ software from the National Institutes of Health.

### Statistical Analysis

All statistical analyses were performed using SPSS22.0 and the figures were created using GraphPad Prism (GraphPad Software Inc.). Data were presented as the mean ± SEM. Comparisons between two groups were made by student 2-tailed unpaired *t*-test. Comparisons between three or four groups were made by one-way ANOVA. *p* value < 0.05 was considered statistically significant (*p < 0.05, **p < 0.01, ***p < 0.001).

## Results

### SRC-1 Expression in APP/PS1 Mice

**Figure 1:**
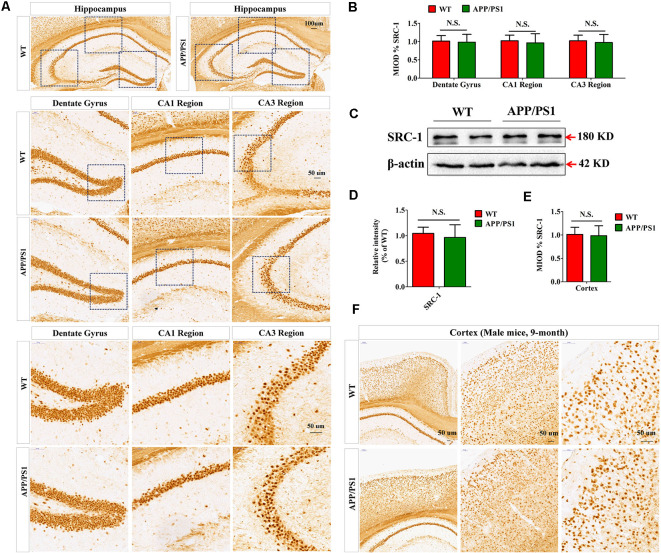
The expression of Steroid receptor coactivator 1 (SRC-1) protein in APP/PS1 mice. **(A,B)** SRC-1 expression in the coronal sections of the hippocampus (10×) in APP/PS1 mice at 8–9 months of age. Dentate gyrus (20× and 40×), CA1 region (20× and 40×), and CA3 region (20× and 40×). **(C,D)** Representative immuno-blot and densitometry analysis of SRC-1 expression in the hippocampus of APP/PS1 mice. **(E,F)** SRC-1 expression in the coronal sections of the cortex (10×, 20× and 40×). Data are presented as the mean ± SEM of six mice in each group. N.S., no significance.

APP/PS1 mice, which express human mutant APP and PS1 (Garcia-Alloza et al., 2006), are commonly used as an Aβ-induced AD model. We first examined the expression of SRC-1 in the hippocampus (Figure 1A) and cortex (Figure 1F) of APP/PS1 mice. Both IHC (Figures 1A,B) and western (Figures 1C,D) results showed that SRC-1 is abundant in the region of the hippocampus but no changed expression was observed in the APP/PS1 mice compared to the littermate WT mice.

### SRC-1 Knockout Shows No Effect on Synaptic Protein Expression in APP/PS1 Mice

In order to observe the possible role of SRC-1 in the AD model, we bred SRC-1^−/−^ mice with APP/PS1 mice and obtained APP/PS1×SRC-1^−/−^ mice at the F2 generation, with the control APP/PS1×SRC-1^+/+^ (Supplementary Figure S1). The immunofluorescent staining of SRC-1 in WT (SRC-1^+/+^), APP/PS1×SRC-1^+/+^ and APP/PS1×SRC-1^−/−^ mice (Supplementary Figure S2) showed no positive staining of SRC-1 in APP/PS1×SRC-1^−/−^ mice, and non-changed SRC-1 expression between the WT and APP/PS1×SRC-1^+/+^ mice, which is consistent with the result in Figure 1.

**Figure 2:**
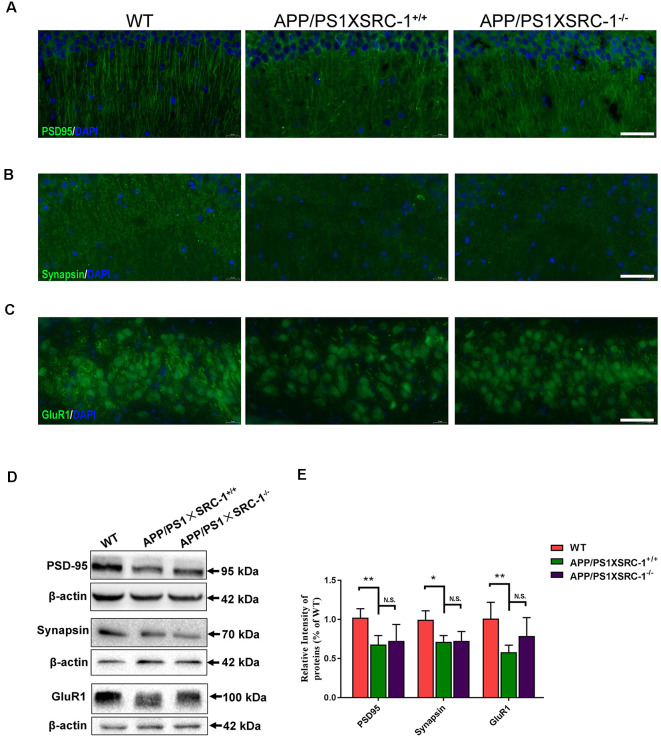
Expression of PSD95, Synapsin and GluR1 in the hippocampus of APP/PS1×SRC-1^−/−^ mice.** (A–C)** The coronal sections of the hippocampus were stained for PSD95 **(A)**, Synapsin **(B)**, GluR1 **(C)**, and 4’,6-diamidino-2-phenylindole (DAPI) in APP/PS1×SRC-1^−/−^, APP/PS1×SRC-1^+/+^ control and wildtype (WT) mice (8–9 months age). Scale bars: 50 μm. **(D,E)** Representative immunoblots and densitometry analysis of PSD95, Synapsin and GluR1 expressions in each group. Data are presented as the mean ± SEM of six mice in each group. *p < 0.05, **p < 0.01 and N.S., no significance.

Synaptic loss is the main reason of the cause cognitive deficiency in AD. Postsynaptic density (PSD) 95, Synapsin and glutamate receptor 1 (GluR1) are the important proteins in synapse and their level shows a positive correlation to synaptic function. The expression of the three selected proteins was detected in the hippocampus of APP/PS1×SRC-1^−/−^ mice (Figure 2). The results showed, compared to the WT mice, that the APP/PS1 (shown as APP/PS1×SRC-1^+/+^) mice exhibited a notable decrease in the expression of PSD95, Synapsin, and GluR1 (Figure 2). However, none of the three synaptic proteins showed any changed expression in the hippocampus of APP/PS1×SRC-1^−/−^ mice, compared to the APP/PS1×SRC-1^+/+^ mice. These findings indicates that the deletion of SRC-1 has no effect on synaptic protein expression in the AD mouse model. Similarly, in the non-AD model (as shown in Supplementary Figure S3), PSD95, Synapsin, and GluR1 expression remain constant in the SRC-1 knockout (SRC-1^−/−^) mice compared to their WT littermates.

### SRC-1 Knockout Exerts No Effect on Aβ Deposition in APP/PS1 Mice

**Figure 3:**
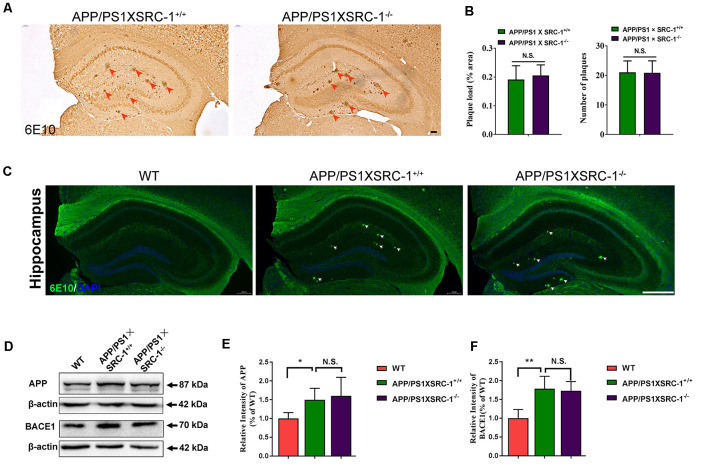
The Aβ plaque in the hippocampus of APP/PS1×SRC-1^−/−^ mice.** (A,C)** The coronal sections of the hippocampus in APP/PS1 with (APP/PS1×SRC-1^+/+^) or without SRC-1 APP/PS1×SRC-1^−/−^), mice of 8–9 months age were stained for Aβ (6E10). Scale bars: 100 μm. **(B)** The mean Aβ plaque load (% area) and number were quantified. **(D–F)** Representative immune-blot and densitometry analysis of APP and BACE-1 expression in the APP/PS1×SRC-1^−/−^, APP/PS1×SRC-1^+/+^ control and WT. Data are presented as the mean ± SEM of six mice in each group. *p < 0.05, **p < 0.01 and N.S., no significance.

The Aβ senile plaque is the major pathological hallmark of AD. To further determine the effects of SRC-1 knockout on AD, we detected the Aβ plaque in both APP/PS1×SRC-1^+/+^ and APP/PS1×SRC-1^−/−^ mice (Figures 3A,C). The IHC results demonstrated that the number and area of the Aβ plaque in APP/PS1×SRC-1^−/−^ mice was comparable to those in APP/PS1×SRC-1^+/+^ mice (Figures 3A,B). It is known that Aβ production is affected by protein levels of APP and APP processing enzymes such as BACE-1. We discovered that APP/PS1×SRC-1^+/+^ mice showed an increased expression of both APP and BACE-1 compared to WT mice (Figures 3D–F); but APP/PS1×SRC-1^−/−^ mice showed an expression change of neither APP nor BACE-1 compared to APP/PS1×SRC-1^+/+^ mice (Figures 3D–F). In addition, we also detected an APP and BACE-1 level in the non-transgenic AD mice and the result showed no expression difference of APP or BACE-1 between SRC-1^−/−^ mice and their WT littermates (Supplementary Figure S4).

### SRC-1 Knockout Exerts No Effect on the Activation of Microglia and Astrocytes in APP/PS1 Mice

**Figure 4:**
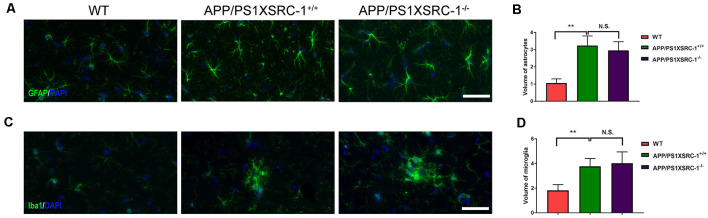
The activation of microglia and astrocyte in the hippocampus of APP/PS1×SRC-1^−/−^ mice.** (A,C)** The coronal sections of the hippocampus in each group were stained for GFAP **(A)**, Iba-1 **(C)** and DAPI. Scale bars: 50 μm. **(B,D)** The volume of astrocyte **(B)** and microglia **(D)** in APP/PS1×SRC-1^−/−^ mice, respectively. Data are presented as the mean ± SEM of six mice in each group. **p < 0.01 and N.S., no significance.

Neuroinflammation characterized by the activation of astrocytes and microglia is also a significant contributor to the pathological progression of AD (Zheng et al., 2018). To investigate whether SRC-1 is involved in the neuroinflammation of APP/PS1 mice, we then stained the coronal sections of the hippocampus of each group of mice with antibodies against GFAP (a marker for astrocytes) or Iba-1 (a marker for microglia; Figures 4A,C). Over activation of glial cells was shown in APP/PS1×SRC-1^+/+^ mice in contrast to WT mice, however, the volume of both astrocytes and microglia in the hippocampus of APP/PS1×SRC-1^−/−^ is similar to that of APP/PS1×SRC-1^+/+^ mice (Figures 4B,D). This result showed that SRC-1 deletion would not affect the over activation status of microglia and astrocytes in the APP/PS1 mice.

## Discussion

Steroids modulate the structure and function of the brain by binding to the NRs (Bayele, 2019; Heck et al., 2020), then the activated NRs recruit the steroid receptor coactivators (SRCs, also named nuclear receptor coactivators) to enhance their transcriptional activities (McEwen and Milner, 2017). SRC-1 is the key coactivator for the transcriptional regulation of NRs (Bayele, 2019; Heck et al., 2020). There are studies that have reported that SRC-1 is widely expressed in the brain (Chen et al., 2015; Kerver and Wade, 2015) and modulates many brain functions, such as motor, energy homeostasis, and reproductive behavior (Charlier et al., 2005; Yang et al., 2019) through the effect on cerebellar Purkinje cells (Eijun et al., 2003), hypothalamic neurons (Yang et al., 2019; Heck et al., 2020), and so on. Furthermore, a recent study has shown that SRC-1 is highly expressed in the hippocampus, and hippocampal SRC-1 knockdown mediated by the lentivirus could cause decreased synaptic protein expression and memory impairment (Bian et al., 2018; Chen et al., 2020). On the other hand, the ER regulates the synaptic protein expression, such as PSD95 and Synapsin, and the cognitive function through the SRC-1-dependent pathway (Liu et al., 2015; Zhao et al., 2017, 2018). This evidence suggests that SRC-1 is implicated in the cognitive function under the physiological condition and might even play a role in brain disorders with defects of cognition.

AD is a neurodegenerative disease and its major symptom is impaired cognitive function. It is of interest to see whether the SRC-1 protein level correlates with AD. In the present study, we first examined the SRC-1 protein level in the hippocampus and cortex of APP/PS1 mice, and the result showed no difference in SRC-1 expression between AD mice and the WT littermates. This result indicates that the transgene of APP/PS1 and the consequent pathology, have no effect on SRC-1 expression. Furthermore, we investigated whether SRC-1 knockout affected the expression of the synaptic protein in AD mice. Our results demonstrated that SRC-1 deficiency (APP/PS1×SRC-1^−/−^) had no effect on the expression of PSD95, Synapsin, and the GluR1 protein in AD. Consistently, the level of the three synaptic proteins in the SRC-1 KO mice was comparable to those in the WT mice.

However, a previous study has reported that SRC-1 knockdown in the hippocampus of mice during adulthood leads to the decreased expression of PSD95 and other synaptic proteins for weeks (Bian et al., 2018; Chen et al., 2020). The discrepancies between their studies and ours might be explained by the different gene manipulations of SRC-1. SRC-1 knockout is the complete deletion of SRC-1 from the embryo period, so its function might be compensated by other coactivators, such as SRC-2 or SRC-3. Another study has actually revealed that knockout of both SRC-1 and SRC-3 during the embryo stage would cause a cardiomyopathy phenotype, however, the heart morphology and tissue structures in mice with either SRC-1 or SRC-3 knockout were very similar to those of WT mice (Chen et al., 2015). Similarly, the same reason might be used to explain that SRC-1 knockdown in adulthood caused the memory loss (Bian et al., 2018) but the SRC-1 knockout did not (Eijun et al., 2003).

The Aβ plaque is the major pathological feature in AD (Masters and Beyreuther, 2006). A decrease of synaptic protein expression in the APP/PS1 mice is probably the result of the overproduction of Aβ (Evin and Weidemann, 2002). Aβ peptides are generated from APP sequentially cleaving by β- and then γ-secretase (Maia and Sousa, 2019; Zhang et al., 2020). In our study, SRC-1 deletion has not affected the Aβ plaque in the APP/PS1 mice, in neither the number nor the area of the plaque load. We also demonstrated that APP/PS1×SRC-1^−/−^ mice showed no change of the expression of the APP and BACE-1 protein. Futhermore, SRC-1^−/−^ mice also exhibited the non-changed expression of APP or BACE-1, similar to that in the AD model. As the source of Aβ, APP and BACE-1 expression was not affected by SRC-1 deletion, which would sustain the result of the invariable Aβ plaque in APP/PS1×SRC-1^−/−^ mice, at least to some extent. Aβ deposition is often accompanied by activation of microglia and astrocytes, an important event in AD brain, which produces proinflammatory cytokines and chemokines causing neuronal dysfunction and further neurodegeneration (Galea et al., 2015). Here, the cell number of astrocytes and microglia increased in the hippocampus of APP/PS1 mice compared to the WT, consistent with previous reports (Lu et al., 2019); but in the APP/PS1×SRC-1^−/−^ group, no clear changes of astrocytes and microglia were observed compared to the APP/PS1×SRC-1^+/+^ group.

Altogether, our results suggest that the SRC-1 knockout would neither change the synaptic protein expression, nor affect the Aβ plaque or activation of astrocytes and microglia in APP/PS1 mice. But this outcome could not completely eliminate the possible role of SRC-1 in AD. First, as we have discussed, SRC-1 knockout and knockdown shows different consequences. SRC-1 knockdown during the adulthood of APP/PS1 mice might possibly exhibit quite different results from this present study. Second, our experiments only detected the effect of SRC-1 deletion, but not the effects of SRC-1 over-expression or activation. Wang et al. (2015) has reported that MCB613 could be a potent small molecule “stimulator” for SRC-1, which could be used to enhance the activity of SRC-1 in APP/PS1 mice for further investigation of the role of SRC-1. Finally, we could not completely exclude the possible connection between SRC-1 and tau hyperphosphorylation, and more experiments are required to further address this question. In summary, our study confirms that SRC-1 knockout has no effect on some of the pathologic features of APP/PS1 mice.

## Data Availability Statement

The datasets generated for this study are available on request to the corresponding author.

## Ethics Statement

The animal study including all experimental and animal protocols were reviewed and approved by the animal studies committees of Dalian Medical University (ethics committee approval permit no. L2013011).

## Author Contributions

SL, Y-HY and J-YY contributed to the conception and design of the project. QW, XZ, NL and MN contributed to the conduct of the experiments and analysis of data. BW wrote the manuscript. Q-FL, D-DZ and RJ contributed to the conduct of the experiments and analysis of data during the revision. QW and X-FW helped with the manuscript revision and proofreading. All authors contributed to the article and approved the submitted version.

## Conflict of Interest

The authors declare that the research was conducted in the absence of any commercial or financial relationships that could be construed as a potential conflict of interest.

## References

[B1] Bayele H. K. (2019). A conserved mechanism of sirtu in signalling through steroid hormone receptors. Biosci. Rep. 39:BSR20193535. 10.1042/bsr20193535 31746335 PMC6904774

[B2] Bian C. Huang Y. Zhu H. Zhao Y. Zhao J. Zhang J. (2018). Steroid receptor coactivator-1 knockdown decreases synaptic plasticity and impairs spatial memory in the hippocampus of mice. Neuroscience 377, 114–125. 10.1016/j.neuroscience.2018.02.034 29524638

[B3] Bian C. Zhu K. Yang L. Lin S. Li S. Su B.. (2012). Gonadectomy differentially regulates steroid receptor coactivator-1 and synaptic proteins in the hippocampus of adult female and male C57BL/6 mice. Synapse 66, 849–857. 10.1002/syn.21574 22623226

[B5] Charlier T. D. Ball G. F. Balthazart J. (2005). Inhibition of steroid receptor coactivator-1 blocks estrogen and androgen action on male sex behavior and associated brain plasticity. J. Neurosci. 25, 906–913. 10.1523/JNEUROSCI.3533-04.2005 15673671 PMC6725610

[B27] Charlier T. D. Seredynski A. L. Niessen N.-A. Balthazart J. (2013). Modulation of testosterone-dependent male sexual behavior and the associated neuroplasticity. Gen. Comp. Endocrinol. 190, 24–33. 10.1016/j.ygcen.2013.03.003 23523709 PMC4761263

[B6] Chen X. Qin L. Liu Z. Liao L. Martin J. F. Xu J. (2015). Knockout of SRC-1 and SRC-3 in mice decreases cardiomyocyte proliferation and causes a noncompaction cardiomyopathy phenotype. Int. J. Biol. Sci. 11, 1056–1072. 10.7150/ijbs.12408 26221073 PMC4515817

[B7] Chen X. Tian Y. Zhu H. Bian C. Li M. (2020). Inhibition of steroid receptor coactivator-1 in the hippocampus impairs the consolidation and reconsolidation of contextual fear memory in mice. Life Sci. 245:117386. 10.1016/j.lfs.2020.117386 32006528

[B8] Eijun N. Yoshida-Komiya H. Chan C.-S. Liao L. Davis R. L. O’Malley B. W.. (2003). SRC-1 null mice exhibit moderate motor dysfunction and delayed development of cerebellar purkinje cells. J. Neurosci. 23, 213–222. 10.1523/JNEUROSCI.23-01-00213.2003 12514218 PMC6742154

[B10] Evin G. Weidemann A. (2002). Biogenesis and metabolism of Alzheimer’s disease aβ amyloid peptides. Peptides 23, 1285–1297. 10.1016/s0196-9781(02)00063-3 12128085

[B11] Fernandez C. G. Hamby M. E. McReynolds M. L. Ray W. J. (2019). The role of APOE4 in disrupting the homeostatic functions of astrocytes and microglia in aging and Alzheimer’s disease. Front. Aging Neurosci. 10:14. 10.3389/fnagi.2019.00014 30804776 PMC6378415

[B12] Galea E. Morrison W. Hudry E. Arbel-Ornath M. Bacskai B. J. Gomez-Isla T.. (2015). Topological analyses in APP/PS1 mice reveal that astrocytes do not migrate to amyloid-β plaques. Proc. Natl. Acad. Sci. U S A 112, 15556–15561. 10.1073/pnas.1516779112 26644572 PMC4697430

[B24] Garcia-Alloza M. Robbins E. M. Zhang-Nunes S. X. Purcell S. M. Betensky R. A. Raju S.. (2006). Characterization of amyloid deposition in the APPswe/PS1dE9 mouse model of Alzheimer disease. Neurobiol. Dis. 24, 516–524. 10.1016/j.nbd.2006.08.017 17029828

[B23] Hasanpour M. Nourazarian A. Geranmayeh M. H. Nikanfar M. Khaki-Khatibi F. Rahbarghazi R. (2018). The dynamics of neurosteroids and sex-related hormones in the pathogenesis of Alzheimer’s disease. Neuromolecular Med. 20, 215–224. 10.1007/s12017-018-8493-y 29728813

[B13] Heck A. L. Thompson M. K. Uht R. M. Handa R. J. (2020). Sex-dependent mechanisms of glucocorticoid regulation of the mouse hypothalamic corticotropin-releasing hormone gene. Endocrinology 161:bqz012. 10.1210/endocr/bqz012 31754709 PMC7188085

[B15] Kerver H. N. Wade J. (2015). Hormonal regulation of steroid receptor coactivator-1 mRNA in the male and female green anole brain. J. Neuroendocrinol. 27, 223–233. 10.1111/jne.12249 25557947

[B16] Li Q. Wu X. Na X. Ge B. Wu Q. Guo X.. (2019). Impaired cognitive function and altered hippocampal synaptic plasticity in mice lacking dermatan sulfotransferase Chst14/D4st1. Front. Mol. Neurosci. 12:26. 10.3389/fnmol.2019.00026 30853887 PMC6396735

[B17] Liu M. Huang X. Zhao Y. Zhang D. Zhang J. (2015). Steroid receptor coactivator-1 mediates letrozole induced downregulation of postsynaptic protein PSD-95 in the hippocampus of adult female rats. J. Steroid. Biochem. Mol. Biol. 154, 168–175. 10.1016/j.jsbmb.2015.07.011 26223010

[B18] Lu Y. Tan L. Wang X. (2019). Circular HDAC9/microRNA-138/Sirtuin-1 pathway mediates synaptic and amyloid precursor protein processing deficits in Alzheimer’s disease. Neurosci. Bull. 35, 877–888. 10.1007/s12264-019-00361-0 30887246 PMC6754481

[B19] Masters C. L. Beyreuther K. (2006). Alzheimer’s centennial legacy: prospects for rational therapeutic intervention targeting the Aβ amyloid pathway. Brain 129, 2823–2839. 10.1093/brain/awl251 17012295

[B22] Maia M. A. Sousa E. (2019). BACE-1 and γ-secretase as therapeutic targets for Alzheimer’s disease. Pharmaceuticals 12:41. 10.3390/ph12010041 30893882 PMC6469197

[B20] McEwen B. S. Milner T. A. (2017). Understanding the broad influence of sex hormones and sex differences in the brain. J. Neurosci. Res. 95, 24–39. 10.1002/jnr.23809 27870427 PMC5120618

[B21] Meng Y. Wang R. Yang F. Ji Z.-J. Fang L. Sheng S.-L. (2010). Amyloid precursor protein 17-mer peptide ameliorates hippocampal neurodegeneration in ovariectomized rats. Neurosci. Lett. 468, 173–177. 10.1016/j.neulet.2009.07.058 19632300

[B25] Qiu L. Zhao Y. Guo Q. Zhang Y. He L. Li W.. (2016). Dose-dependent regulation of steroid receptor coactivator-1 and steroid receptors by testosterone propionate in the hippocampus of adult male mice. J. Steroid. Biochem. Mol. Biol. 156, 23–31. 10.1016/j.jsbmb.2015.11.012 26607693

[B14] Ross J. L. Kushner H. Kowal K. Bardsley M. Davis S. Reiss A. L.. (2017). Androgen treatment effects on motor function, cognition, and behavior in boys with klinefelter syndrome. J. Pediatr. 185, 193.e4–199.e4. 10.1016/j.jpeds.2017.02.036 28285751 PMC6754744

[B26] Tang Y. Min Z. Xiang X. J. Liu L. Ma Y. L. Zhu B. L.. (2018). Estrogen-related receptor α is involved in Alzheimer’s disease-like pathology. Exp. Neurol. 305, 89–96. 10.1016/j.expneurol.2018.04.003 29641978

[B28] Villemagne V. L. Burnham S. Bourgeat P. Brown B. Ellis K. A. Salvado O.. (2013). Amyloid β deposition, neurodegeneration, and cognitive decline in sporadic Alzheimer’s disease: a prospective cohort study. Lancet Neurol. 12, 357–367. 10.1016/S1474-4422(13)70044-9 23477989

[B4] Wang B. Wu Q. Lei L. Sun H. Michael N. Zhang X.. (2018). Long-term social isolation inhibits autophagy activation, induces postsynaptic dysfunctions and impairs spatial memory. Exp. Neurol. 311, 213–224. 10.1016/j.expneurol.2018.09.009 30219732

[B29] Wang L. Yu Y. Chow D. C. Yan F. Hsu C. C. Stossi F.. (2015). Characterization of a steroid receptor coactivator small molecule stimulator that overstimulates cancer cells and leads to cell stress and death. Cancer Cell. 28, 240–252. 10.1016/j.ccell.2015.07.005 26267537 PMC4536575

[B30] Wattmo C. Wallin A. K. (2017). Early- versus late-onset Alzheimer’s disease in clinical practice: cognitive and global outcomes over 3 years. Alzheimers Res. Ther. 9:70. 10.1186/s13195-017-0294-2 28859660 PMC5580278

[B31] Yang Y. van der Klaauw A. A. Zhu L. Cacciottolo T. M. He Y. Stadler L. K. J.. (2019). Steroid receptor coactivator-1 modulates the function of pomc neurons and energy homeostasis. Nat. Commun. 10:1718. 10.1038/s41467-019-08737-6 30979869 PMC6461669

[B32] Zalachoras I. Verhoeve S. L. Toonen L. J. van Weert L. T. van Vlodrop A. M. Mol I. M.. (2016). Isoform switching of steroid receptor co-activator-1 attenuates glucocorticoid-induced anxiogenic amygdala CRH expression. Mol. Psychiatry 21, 1733–1739. 10.1038/mp.2016.16 26976039

[B34] Zhang D. Guo Q. Bian C. Zhang J. Lin S. Su B. (2011). Alterations of steroid receptor coactivator-1 (SRC-1) immunoreactivities in specific brain regions of young and middle-aged female Sprague–Dawley rats. Brain Res. 1382, 88–97. 10.1016/j.brainres.2011.01.024 21241680

[B33] Zhang X. Zhao F. Wang C. Zhang J. Bai Y. Zhou F.. (2020). AVP(4–8) improves cognitive behaviors and hippocampal synaptic plasticity in the APP/PS1 mouse model of Alzheimer’s disease. Neurosci. Bull. 36, 254–262. 10.1007/s12264-019-00434-0 31605298 PMC7056786

[B35] Zhao J. Bian C. Liu M. Zhao Y. Sun T. Xing F.. (2018). Orchiectomy and letrozole differentially regulate synaptic plasticity and spatial memory in a manner that is mediated by SRC-1 in the hippocampus of male mice. J. Steroid. Biochem. Mol. Biol. 178, 354–368. 10.1016/j.jsbmb.2018.02.007 29452160

[B37] Zhao Y. He L. Zhang Y. Zhao J. Liu Z. Xing F. Z.. (2017). Estrogen receptor α and β regulate actin polymerization and spatial memory through an SRC-1/mTORC2-dependent pathway in the hippocampus of female mice. J. Steroid. Biochem. Mol. Biol. 174, 96–113. 10.1016/j.jsbmb.2017.08.003 28789972

[B36] Zhao L. Woody S. K. Chhibber A. (2015). Estrogen receptor β in Alzheimer’s disease: from mechanisms to therapeutics. Ageing Res. Rev. 24, 178–190. 10.1016/j.arr.2015.08.001 26307455 PMC4661108

[B38] Zheng H. Cheng B. Li Y. Li X. Chen X. Zhang Y. W. (2018). TREM2 in Alzheimer’s disease: microglial survival and energy metabolism. Front. Aging Neurosci. 10:395. 10.3389/fnagi.2018.00395 30532704 PMC6265312

