# Supplementary material for: SRC-1 Knockout Exerts No Effect on Amyloid β Deposition in APP/PS1 Mice
Source: Front Aging Neurosci. 2020 Jun 17;12:145. doi: 10.3389/fnagi.2020.00145 (PMC7311769; doi:10.3389/fnagi.2020.00145)
Supplement: Supplementary file 1 [file Data_Sheet_1.pdf]

Supplementary Material

Materials and Methods

Genotyping of SRC-1 KO mice and APP/PS1 mice

Genotyping of the SRC-1 mutant was performed using two primer pairs. The single-band appearing at the 300bp mark represents wild type (SRC-$1^{+/+}$) mice, whereas a single band appearing at the 600bp is homozygote (SRC-$1^{-/-}$). The appearance of both bands represents SRC-1 heterozygote (SRC-$1^{+/-}$).

Genotyping of APP/PS1 was performed using primers below to detect APP and PS1. The PCR amplification was performed using the following conditions: one cycle of 94°C for 3 min and 30 cycles of 94°C for 30 s, 56°C for 30 s, and 72°C for 40 s, and then 72°C for 10 min. Bands appearing for the two primers were APP/PS1 mice, whereas no appearance of any band represents wildtype mice.

For APP: 5’-GAC,TGA,CCA,CTC,GAC,CAG,GTT,CTG

5’-CTT,GTA,AGT,TGG,ATT,CTC,ATA,TCC,G

For PS1: 5’-AAT,AGA,GAA,CGG,CAG,GAG,CA

5’-GCC,ATG,AGG,GCA,CTA,ATC,AT

Genotyping of APP/PS1×SRC-1-/- was performed by the detection of APP/PS1 and the SRC mutant, respectively. PCR results were reconfirmed by western-blot (Fig. S5).

Supplementary Figures

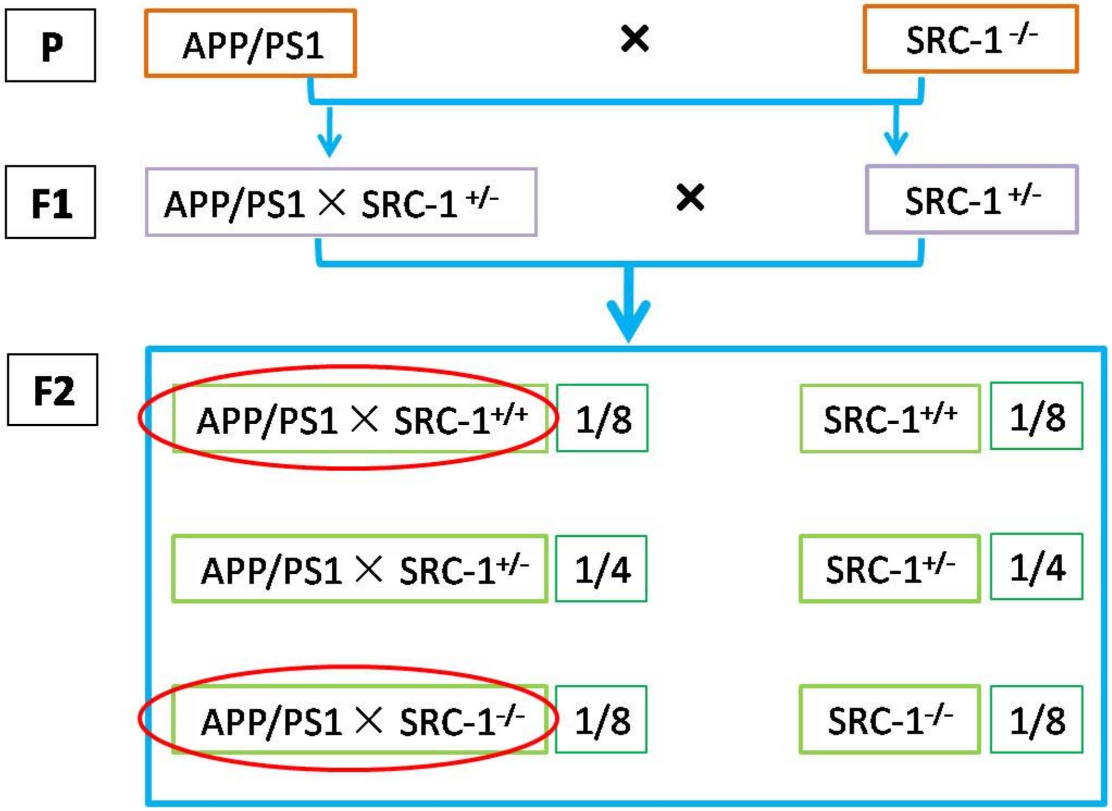

Figure S1

Breeding scheme of the APP/PS1×SRC-1$^{-/-}$ mice. The breeding of APP/PS1 and SRC-1 KO mice from the parent generation (P) to the F2 generation. In the P, the APP/PS1 mouse and the SRC-1 KO mouse were either male or female in each genotype. In F1, all the mice were SRC-1 heterozygotes (+/-) with or without the APP/PS1 gene. In F2, the red circle exhibits the mice with the desired gene type and all male mice aged 8-9 months were used in the experiments.

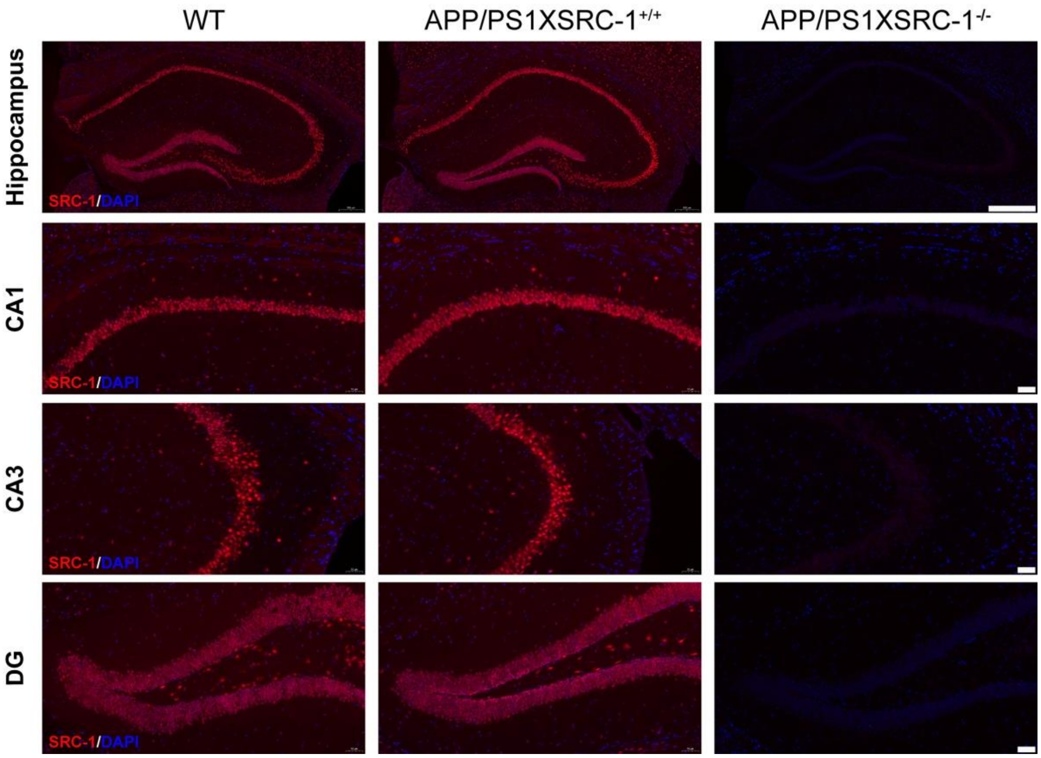

Figure S2.

Expression of SRC-1 in the hippocampus of APP/PS1×SRC-1$^{-/-}$ mice. The coronal sections of the hippocampus were immunostained with SRC-1 (red) antibody in APP/PS1×SRC-1$^{-/-}$, control APP/PS1×SRC-1$^{+/+}$ and wildtype (WT) mice. DAPI (blue). Scale bars: hippocampus, 400 μm; the region of CA1, CA3 and DG, 50 μm.

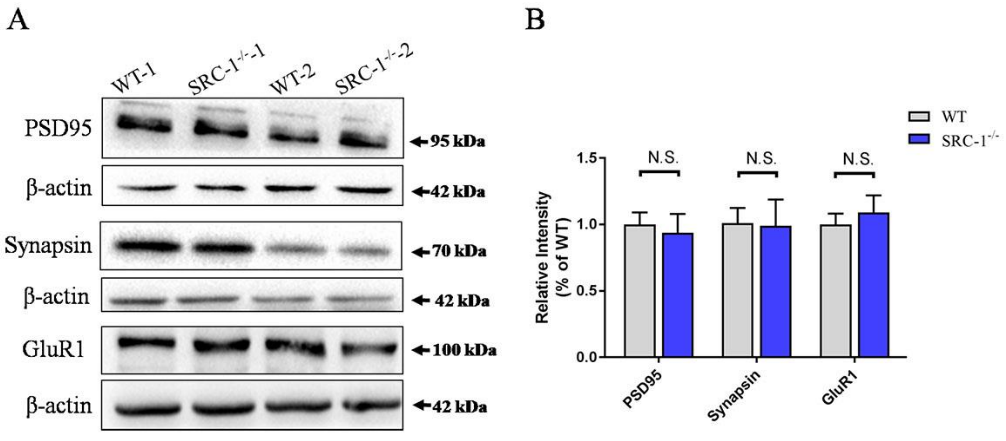

Figure S3.

Expression of synaptic protein in the hippocampus of SRC-1 knockout mice. **A. B:** Representative immuno-blot and densitometry analysis of PSD95, Synapsin and the GluR1 protein expression in the in SRC-1 knockout mice, respectively; Data are presented as the mean ± SEM of six mice in each group. N.S., no significance.

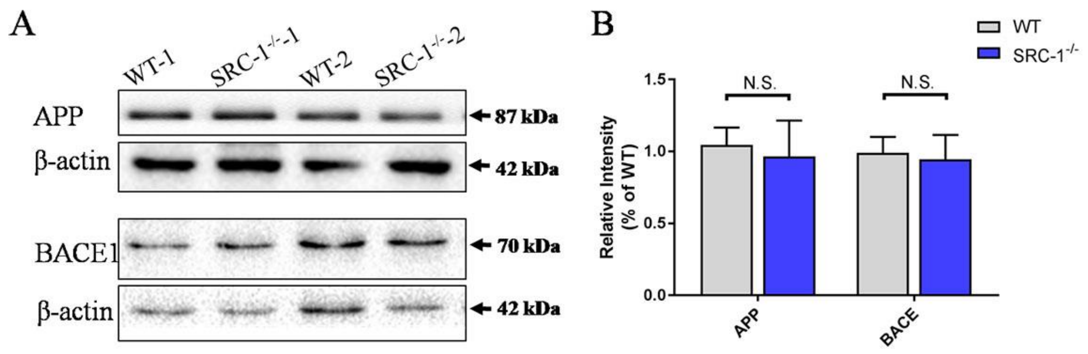

Figure S4.

Expression of APP and BACE-1 in the hippocampus of SRC-1 knockout mice. **A. B:** Representative immuno-blot and densitometry analysis of APP and BACE-1 protein expression in the in SRC-1 knockout mice, respectively; Data are presented as the mean ± SEM of six mice in each group. N.S., no significance.
